# Influence of different polymeric materials of implant and attachment on stress distribution in implant-supported overdentures: a three-dimensional finite element study

**DOI:** 10.1186/s12903-025-05440-5

**Published:** 2025-01-31

**Authors:** Sherif Elsayed, Yousra Ahmed, Mohamed I. El-Anwar, Enas Elddamony, Reem Ashraf

**Affiliations:** 1 Al-Ryada University for Science and Technology, Sadat City, Menoufia Egypt; 2 Department of Prosthetic Dentistry, Removable Prosthodontics Division, Faculty of Dentistry, King Salman International University, El Tur, South Sinai Egypt; 3 Mechanical Engineering Department, National Research Centre (NRC), Dokki, Giza, Egypt; 4 Department of Prosthetic Dentistry, Biomaterials Division, Faculty of Dentistry, King Salman International University, El Tur, South Sinai Egypt

## Abstract

**Purpose:**

Investigating high performance thermoplastic polymers as substitutes to titanium alloy, in fabrication of implants and attachments to support mandibular overdenture, aiming to overcome stress shielding effect of titanium alloy implants.

**Aim of study:**

Assessment of stress distribution in polymeric prosthetic components and bone around polymeric implants, in case of implant-supported mandibular overdenture.

**Materials and methods:**

3D finite element model was established for mandibular overdenture, supported bilaterally by two implants at canine region, and retained by two ball attachments. Linear static stress analysis was carried out by ANSYS 2020 R1. Three identical models were created with different materials for modeling of prosthetic components (implant body, gingival former, ball attachment and matrix). The Monolithic principle was applied as the same material was used in modelling all the prosthetic components in each model (Titanium alloy grade V, poly-ether-ether-ketone (PEEK) and poly-ether-ketone-ketone (PEKK)). Simultaneous Force application of 60 N was carried out bilaterally at the first molar occlusal surface area using 3 runs (vertical, lateral and oblique).

**Results:**

PEEK and PEKK prosthetic components exhibited the highest total deformation and critical Maximum von Mises stresses values in implant body and gingival former under lateral and oblique loads. The stress values approached the fatigue limit of both polymeric materials presenting low factor of safety (< 1.5). The Peri-implant cortical bone in case of PEEK and PEKK showed nearly double maximum principal stresses compared with the titanium model. Conversely, Maximum von Mises stresses in spongy bone were lower in polymeric models than those of titanium ones. Additionally maximum equivalent strain values in spongy peri-implant bone of polymeric models were also lower than those of titanium model.

**Conclusion:**

Critical high stresses were induced in implant body and gingival former under oblique or lateral loadings, accordingly, fatigue failure of both PEEK and PEKK polymer prosthetic elements was estimated due to low factor of safety. Both PEEK and PEKK Polymer models offered no advantage over titanium one regarding stress shielding effect, due to low stress and strain values generated at spongy peri-implant bone in polymer models.

## Background

Fabrication of an implant supported overdenture prosthesis can provide an efficient function and stability of the denture through a variety of factors, such as improved biting force, chewing efficiency. Edentulous mandible can be rehabilitated with two implants-supported overdenture, which is considered a convenient treatment option for completely edentulous patients [1]. Attachment systems used for implant supported overdenture include bars, magnets, balls, and different cylindrical attachment types, fabricated with different materials, concepts and design forms [2]. Attachments not only aid in enhancing the overdenture's retention and stability, but they also have an impact on how much stress is placed on the nearby bone. The success and lifetime of an implant prosthesis are determined by the stress distribution to the alveolar bone [3].

Ball attachments are considered simple and widely used attachment systems as they are more economic and less technique sensitive, having the advantage of being able to distribute and reduce the load transmitted to the implant by allowing slight multidirectional movements [1].

Having different types of implants together with the introduction of new biomaterials, that modify implant properties, had affected positively the influence of implants on the surrounding bone specially when it is related to implant supported prosthesis [4]. To ensure the successful long-term service of the prosthesis, it is crucial to control the stresses to which the load-bearing determinants are exposed. The forces exerted on implants are affected by their magnitude, duration, type, direction and magnification. Additionally, the load transferred to the bone-implant interface is influenced by the biomaterial properties of the implant and the prosthesis, the geometry and surface microstructure of the implant, the nature and quality of the implant-bone interface and the density of bone [5].

In the case of preservation of natural teeth where the periodontal ligament is conserved, the overdenture will dissipate the masticatory forces in a uniform and homogenous pattern, consequently less stress on the prosthetic components would be generated, which can justify the lower incidence of orofacial pain and temporomandibular disorders. But in case of edentulous patients, chewing load dissipation is compromised and is usually accumulated at the interface causing pressure on bone and subsequently failure of the prosthetic appliance [6, 7]. Overload caused by the implant can lead to an increase in rate of bone resorption or fatigue failure of the implant, whereas underloading of the bone may lead to disuse atrophy and subsequent bone loss. Thus, stress distribution is crucial for survivability of the prosthesis [8].

Titanium (Ti) which is considered the gold standard for dental implants, has proven success in terms of rigidity and longevity, but concerns regarding stress shielding and its ability to distribute forces in a pattern that would not affect bone are still questionable. Titanium alloy has an elastic modulus of (110 GPa) compared to bone with elastic modulus (14 GPa), affects the distribution of load over the prosthetic appliance. This was the main reason for introduction of new materials that might have better pattern of stress transfer to the surroundings [9, 10]. Also, recently concerns aroused about toxicity and biocompatibility of titanium or titanium alloy implants [11].

The newly introduced ultra-high performance polymeric materials have attracted interest lately. The polyaryletherketones (PAEKs) family, thermoplastic polymers of ultra-high performance, includes both polyetherketoneketone (PEKK) and polyetheretherketone (PEEK). They are semi-crystalline polymers, known for their superior mechanical performance, which has drawn the attention of researchers and clinicians to explore their use in a variety of dental prosthetic designs, implants, and associated products as a replacement for titanium [12].

PEEK polymer, which is a semicrystalline polyaromatic thermoplastic resin, is known for its high toughness, fatigue, and creep resistance as well as its high thermal stability and erosion resistance. Moreover, the mechanical properties of PEEK are of high resemblance to human bone that may have a great influence on distribution of stresses among the prosthesis-bone interface. One of the main advantages of PEEK is having low weight, good esthetics, and ease of fabrication [13–15]. Some studies suggest that PEEK material, owing to its low elastic modules and high shock-absorbing capacity, is anticipated to evenly distribute the stresses generated during mastication on implants and bone when used as a superstructure framework [16].

Recently, PEKK has been used in dentistry as a prosthetic and implant biomaterial due to suitable fracture resistance, shock-absorbing effect, and favorable stress distribution [17]. It was reported that using PEKK for fabrication of the prostheses framework and overdenture attachments could result in favorable stress distribution with reduction of stresses generated at the supporting implants, however, information about its use as a monolithic implant-prosthesis is still scarce [18].

The finite element analysis (FEA) is a widely used biomechanical evaluation method, used to analyze stress distribution of orthodontic appliances, fixed prostheses, complete dentures, overdentures and dental implants, as well as stress distribution in peri-implant bone and natural or restored teeth [19]. Moreover, Factor of safety (FOS),which is the ratio between yield stress and Maximum von Mises stress generated by FEA, is crucial for estimating the long-term serviceability of prosthetic components such as abutments or implants to predict long-term bone health after implant insertion [19, 20].

Research studies investigating the intraoral performance of PEEK and PEKK implants are limited. However, they are susceptible to aging in saliva due to pH fluctuation that might affect their microhardness and roughness, subsequently the integrity and nature of the biofilm [21, 22].Regarding the exposure of polymer implants to exaggerated stress as in parafunctional habits like bruxism, PEKK implants and attachments may perform better than PEEK ones in bruxer patients due to their higher compressive strength [23, 24]. Furthermore, PEEK fatigue limit is significantly related to the configuration of the cyclic loading since frequency and amplitude of load highly affects fatigue life. PEEK’s fatigue behavior is also significantly different under stress-controlled and strain-controlled conditions during implant applications [25, 26].

Mechanical fatigue life of PEEK implant was tested in different notched specimens in physiological fluid, it was concluded that the fatigue life was greatly affected by notch depth, the greater the depth of notch, the shorter the fatigue life. It was also found that as the notch depth increased, the PEEK specimens spent more time in crack initiation rather than crack propagation, a phenomenon which could cause catastrophic gross failure clinically without apparent structural changes. Accordingly, polymer implant design e.g. dimensions of thread pitch, depth or width can have great influence on fatigue life [26].Thus PAEK family still needs further investigations on the stresses induced upon their use as an implant-prosthesis and the mode of dissipation of these stresses to the surrounding bone.

Therefore, the current study aimed to evaluate stress generated in bone and prosthetic components of implant supported mandibular overdenture using different polymeric materials in comparison to titanium alloy using finite element analysis. Two null hypotheses were assumed in this study. The first was that no difference would exist between the three materials regarding stresses generated in implants or attachments. The second null hypothesis was that stresses induced in peri-implant bone would be the same using the forementioned materials.

## Materials and methods

### Finite element modeling

One finite element model, based on Geng et al. [27], was prepared specially for this study, in order to simulate the clinical situation of an edentulous mandible that was restored with an implant supported overdenture.

The overdenture geometry was acquired by using laser scanner (Geomagic Capture, 3D Systems, Cary, NC, USA). Scanner produced a data file containing a cloud of points coordinates (STL file). An intermediate, software was required (3-Matic version 7.01—Materialise NV, Leuven, Belgium) to trim and create outer surface by the acquired points. Then, the solid (closed) overdenture geometry was exported as IGES file format. This file was imported in an engineering CAD/CAM software "Solidworks" Version 2014 (Dassault Systèmes Inc., 13,090 Aix-en-Provence, France) to eliminate any error that might appear during transforming clouds of points into solid geometry. Finally the solid part (overdenture) was exported as a STEP file format [28].

"Sky classic" regular platform dental implant (bredent medical GmbH & Co. KG—Weissenhorner Str. 2 · 89,250 Senden—Germany) with nominal diameter of 4.0 mm, a length of 12 mm (REF kSKY4012), and a shape of internal Torx® of 2.1 mm was modeled on engineering CAD/CAM software “SolidWorks” Version 2014 (Dassault Systèmes Inc., 13,090 Aix-en-Provence, France), by the help of an implant model offered by the manufacturer.

**Fig. 1:**
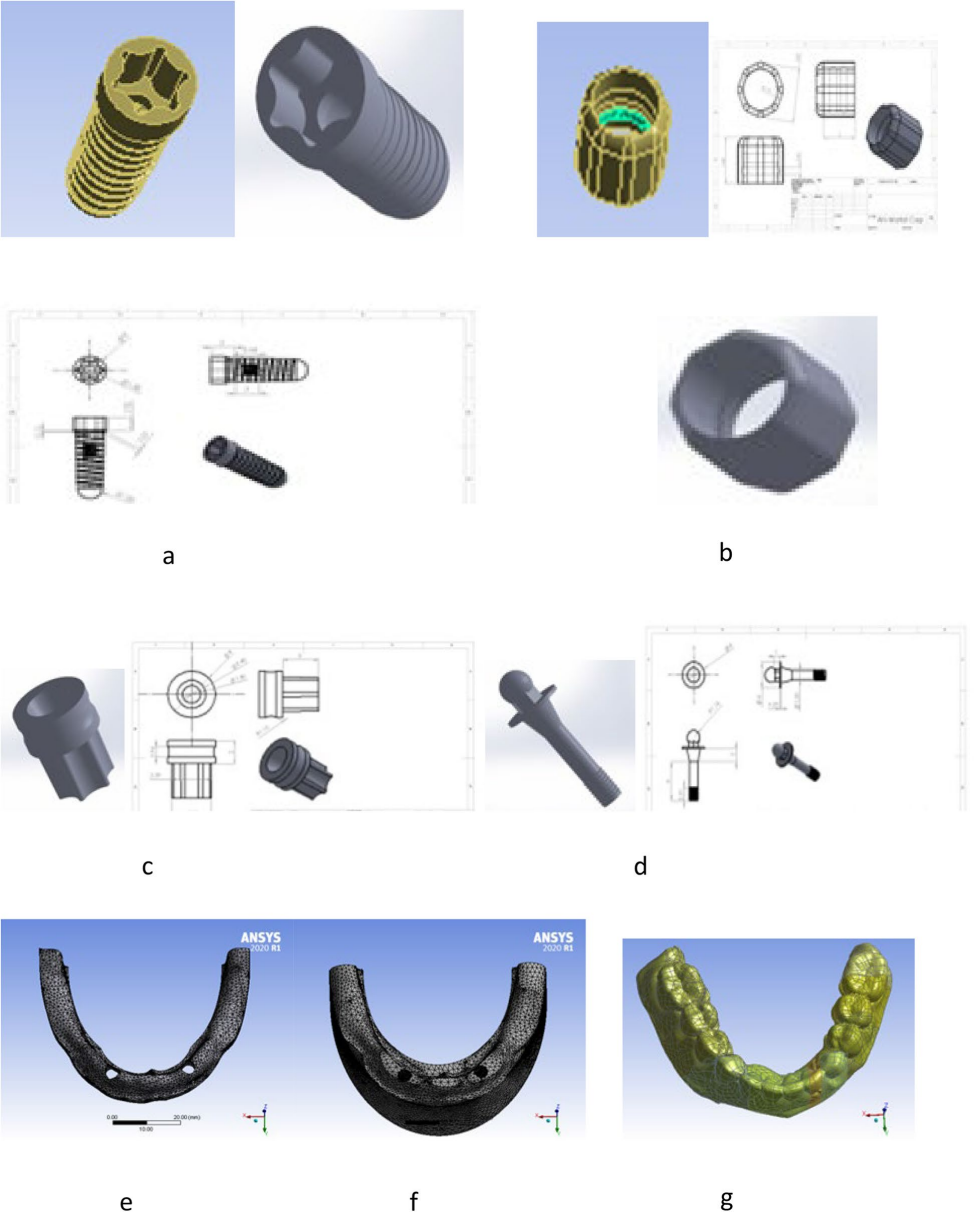
Some modeled components (**a**). implant body, **b** matrix, **c** gingival former, **d** ball attachment, **e** mucosa, **f** cortical bone, **g** acrylic overdenture

Other prosthetic elements related to the same implant system (SKY implant system) like ball attachment (REF SKY-KA02), gingival former, rubber O-ring and the metal matrix (REF SKY-OR50) were also modeled according to the same manufacturer catalogue data. These components were exported as STEP files, to be assembled in the finite element package (ANSYS version 2020 R1, Canonsburg, USA) as shown in Fig. 1.

Two-millimeter thickness layers for mucosa and cortical bone were created by extruding the lower surface of the laser scanned overdenture. Two "U shaped” areas were extruded to create volumes for cortical and cancellous (spongy) bone [29], so that full bond was assumed between cortical bone, spongy bone and mucosa. It was assumed that complete osseointegration was presented between implant and bone. While frictional contact between rubber O-ring from one side and ball attachment / matrix from the other side was 0.3 frictional coefficient. Also, frictional contact between fitting surface of overdenture and mucosa was 0.3 frictional coefficient.

Set of Boolean operations between the modeled components were performed to create cavities for internal components before obtaining the complete model assembled. Meshing of these components was done by 3D brick solid element “Solid-185” [30], which has three degrees of freedom (translations in main axes directions). The resulting numbers of nodes and elements are listed in Table 1.

**Table 1:** Number of nodes and elements for each modeled component

Component	Number of Nodes	Number of Elements
Acrylic Overdenture	425,229	266,944
Mucosa (2 mm in thickness)	42,453	23,614
Matrix	134,596	40,354
Fluro-Rubber O-ring	48,536	28,320
Ball attachment	45,580	26,632
gingival former	157,772	97,382
Implant body	372,394	230,128
Cortical bone (2 mm in thickness)	115,759	71,389
Spongy bone	28,578	17,456

### Materials used for modeling

Three models with the same design were fabricated, representing mandibular overdenture supported bilaterally by two implants in the canine region and retained by two ball attachments, using different materials, Titanium alloy grade V, PEEK and PEKK, for modeling the implant body, gingival former, ball attachment and the housing matrix.

Meanwhile, acrylic resin and plastic-Fluro-rubber were used respectively for modelling overdenture and O-ring in all models. All materials were assumed to be isotropic, homogenous, and linearly elastic and their properties were listed in Table 2.

**Table 2:** Material elastic properties

Material	Young's modules [MPa]	Poisson's ratio	References
Acrylic resin	2,830	0.45	[31]
Mucosa	1	0.37	[32]
Fluro Rubber O-ring	4	0.37	[32]
Titanium	110,000	0.35	[19]
PEEK	3,700	0.40	[31]
PEKK	5,100	0.40	[31, 33]
Cortical bone	14,700	0.30	[31, 34]
Cancellous (spongy) bone	1,470	0.30	[31]

### Constraints and loading conditions

Lower surface of the mandibular cortical bone was set to be fixed in place as boundary condition [35]. Three types of load located on central fossa of first molars (on both sides simultaneously) were tested as; (a) 60 N vertical, (b) 60 N lateral (horizontal) buccolingual, and (c) 60 N Oblique at 45º degree buccolingual.

### Convergence test and analysis

**Fig. 2:**
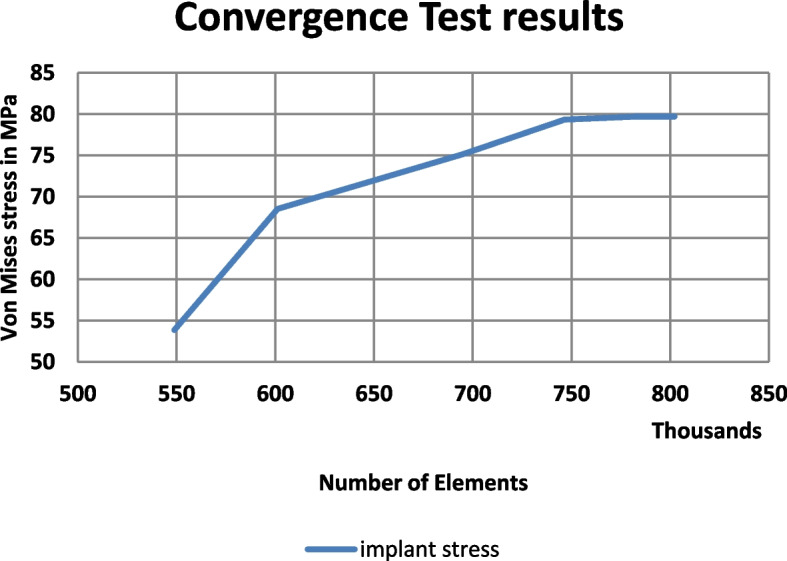
Convergence test graph for implant body meshing

The meshing convergence test was performed by applying test load on different mesh densities, to ensure results accuracy for the discrete model. Initial mesh (coarse one) was examined and its resultant maximum Von Mises stress on implants body was recorded. Running simulations with finer set of meshes were carried out. By comparing the results (Fig. 2), and when no significant difference from one run to another (less than 3%) was recorded, it was indicated that this mesh was fine enough, and the stress results were accurate.

Linear static analysis was performed on Workstation HP Z820 (Dual Intel Xeon E5-2670 v2 processors, 2.5 GHz, 64.0 GB RAM), using commercial multipurpose finite element software package (ANSYS version 2020 R1), the results of these models were verified against similar studies [36].

## Results

A Total of nine runs were performed during this study. As for each model (titanium alloy grade V, PEEK and PEKK) there were three loading scenarios (vertical, lateral and oblique).

### Maximum von Mises stresses and total deformation

Maximum (Max) von Mises stresses values were extracted for all components of each model (titanium alloy, PEEK and PEKK) in the nine runs. Max von Mises stresses values together with images of stress distribution from ANSYS 2020 in the nine runs, were used to evaluate stress distribution in the ductile components (overdenture, mucosa, matrix, rubber O-ring, ball attachment, gingival former, implant body, spongy bone) [20].

Deformation values in the three dimensions (X, Y and Z) were extracted, together with the final total deformation of all components of each model in each run.

**Fig. 3:**
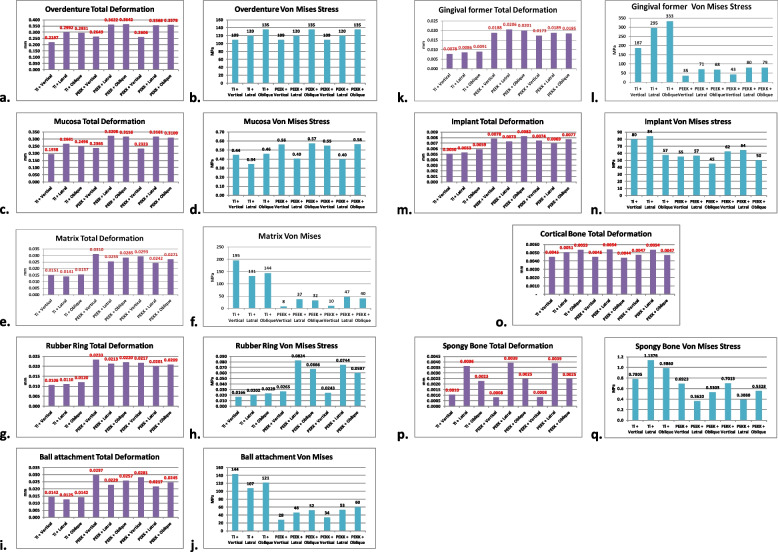
Graphical representation for total deformation in (**a**) overdenture, **c** mucosa, **e** matrix, **g** rubber ring, **i** ball attachment, **k** gingival former, **m** implant, **o** cortical bone, **p** spongy (cancellous) bone, and maximum Von Mises stresses in (**b**) overdenture, **d** mucosa, **f** matrix, **h** rubber ring, **j** ball attachment, **l** gingival former, **n** implant, **q** spongy (cancellous) bone

To extract findings from many results, comparison of extreme values appeared on each of the model components was essential. Thus, graphs in Fig. 3 compared the results with significant differences to be discussed.

### Overdenture

Regarding Max von Mises stresses values in overdenture structure, (Fig. 3b) they were not sensitive to implant and prosthetic elements material. While using titanium prosthetic elements reduced overdenture deformation by about 50–60 microns in comparison to PEEK or PEKK one(s). Polymers (PEEK and PEKK) showed equivalent values for overdenture deformation (around 360 microns in lateral and oblique forces application, 260 microns in vertical force application) (Fig. 3a).

### Mucosa

By comparing models of PEEK and PEKK, mucosa showed nearly similar total deformation results, but both polymer models showed more mucosa deformation by about 40–65 microns in comparison to the titanium model. In addition, Max von Mises stresses of mucosa in polymer models were about 0.1–0.12 MPa higher than corresponding values of titanium model (Figs. 3c,d).

### Matrix

**Fig. 4:**
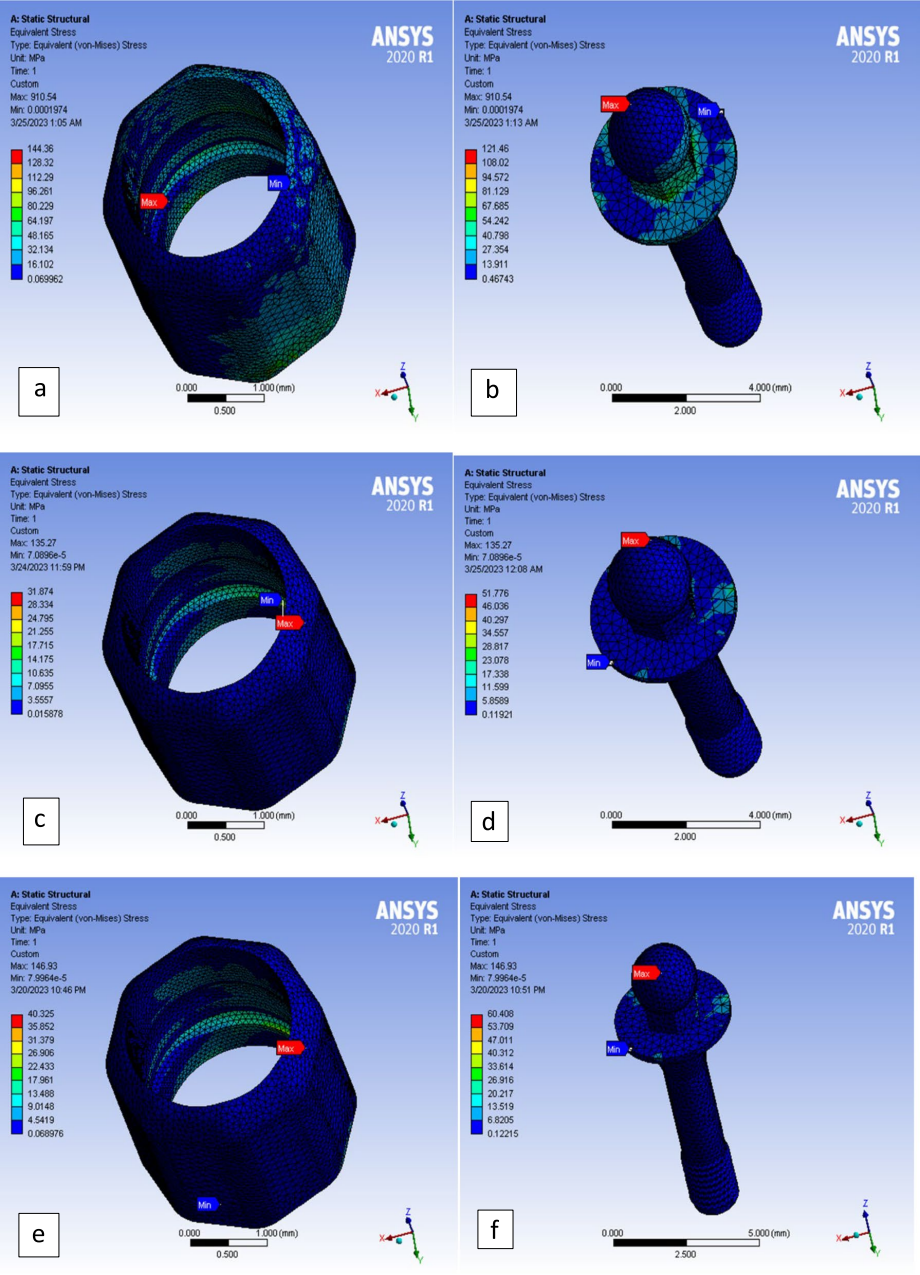
Maximum von Mises stresses under oblique loading in: **a** titanium matrix, **b** titanium ball attachment, **c** PEEK matrix, **d** PEEK ball attachment, **e** PEKK matrix, **f** PEKK ball attachment

PEEK matrices deformed more than PEKK ones with very small differences. Both polymeric matrices (PEEK and PEKK) showed deformation values about double those of titanium matrices (Fig. 3e). Titanium matrices received about 20 times higher Max von Mises stresses values than PEEK and PEKK matrices in case of vertical loading, and 3–4 times higher in case of lateral and oblique loadings (Figs. 3f and 4a,c,e).

### Rubber O-ring

As the most resilient and soft material within this study, it deforms by about 10–12 microns with titanium model and about 20–23 microns with polymeric ones. This trend was extended to stresses by higher ratio. That polymeric rubber rings (PEEK and PEKK) received from about 1.5 times (in case of vertical loading) to about four times (in case of lateral loading) more Max von Mises stresses than those of titanium model. The Max von Mises stresses appeared on rubber ring in all tested model were less than 0.1 MPa (Fig. 3g,h).

### Ball attachments

Metal ball attachments deformed less than polymeric ones. Titanium ball attachments showed deformation values of about 50–55% those of polymeric ones. PEEK ball attachments deformed slightly higher than PEKK ball attachments (Fig. 3i). On other hand, titanium ball attachments showed Max von Mises stresses values much higher than those of polymeric ball attachments. In vertical loading Ti ball attachments showed Max von Mises stresses about 5 times higher than PEEK and 4 times higher than PEKK ones. In lateral and oblique loading Ti ball attachments showed Max von Mises stresses about double or more than those of polymeric ones (Fig. 3j). In all loading scenarios for the three models, the Max von Mises stresses were located at the heads of ball attachments (Fig. 4b,d,f).

### Gingival former

**Fig. 5:**
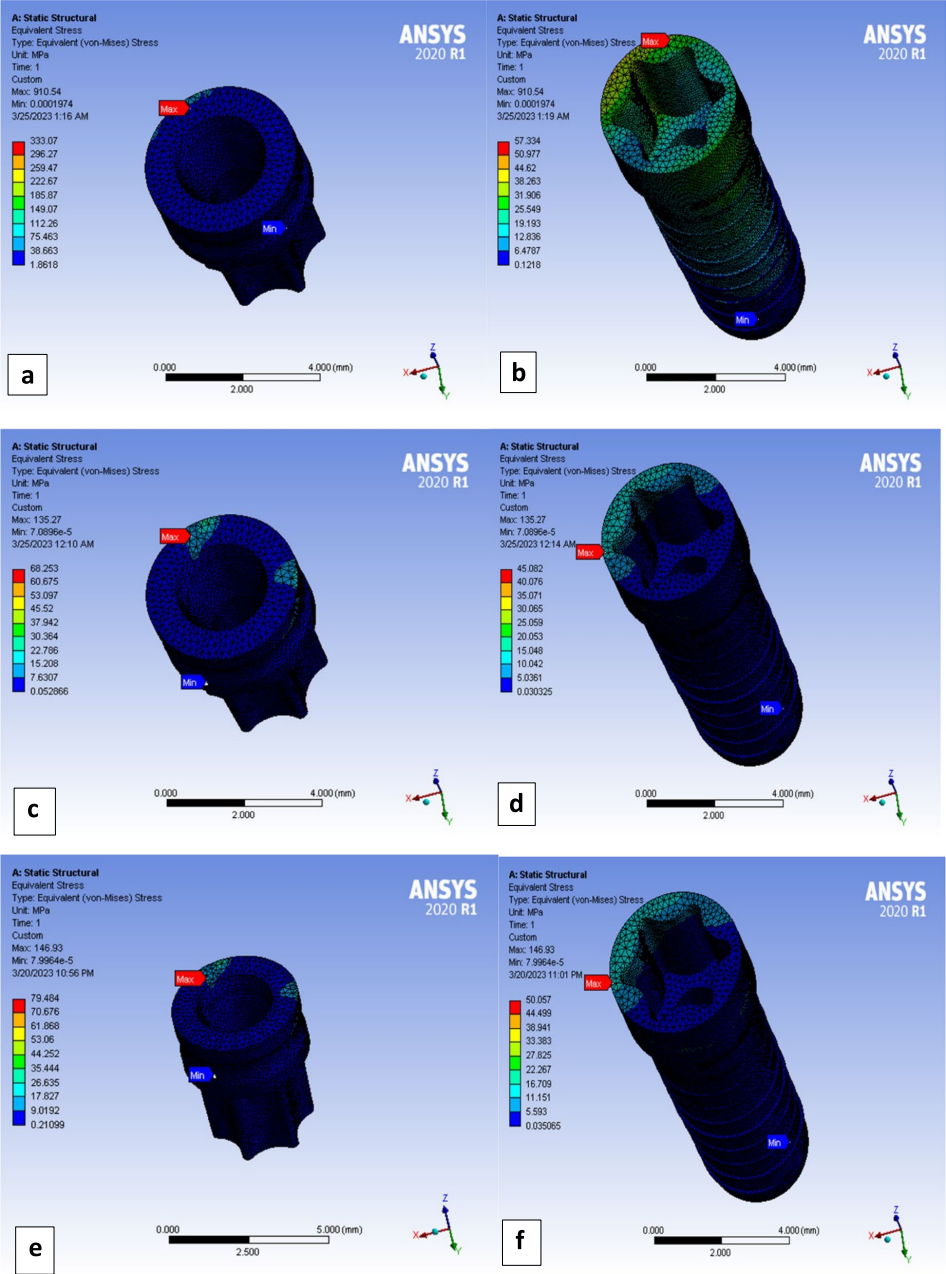
Maximum von Mises stresses under oblique loading in: **a** titanium gingival former, **b** titanium implant, **c** PEEK gingival former, **d** PEEK implant, **e** PEKK gingival former, **f** PEKK implant

Titanium alloy gingival former showed deformation less than 50% that of polymeric one(s) in the three loading scenarios. On the other hand, after vertical loading Ti gingival former received about 5 times higher values of Max von Mises stresses in comparison to PEEK one(s), and 4 times higher than PEKK ones. After lateral loading Ti gingival former received about 4 times higher values of Max von Mises stresses in comparison to PEEK one(s), and more than 3 times higher than PEKK ones. Finally on oblique loading Ti gingival former received *highest Max von Mises stresses value in this study* (333 MPa), with more than 4 times higher than those of PEEK and PEKK ones. (Fig. 3k and l). In all loading scenarios for the three models, the Max von Mises stresses were located at gingival former upper surface (platform) (Fig. 5a,c,e).

### Implant body

Regarding polymeric implant bodies (PEEK or PEKK), the lowest deformation values were evident with lateral load, but deformation increased under vertical load and was highest with oblique loading. In the case of Titanium alloy implant body, it showed lowest total deformation in vertical loading and highest value of deformation with oblique loading. By comparing models to each other, titanium implants received less deformation in comparison to polymeric ones (metal implants showed about 65–75% those of polymeric implants’ deformation values) (Fig. 3m). On the other hand, titanium implant received about 20–30 MPa more Max von Mises stresses in comparison to polymeric one(s), except in case of oblique loading, where the stresses values were near to each other among the three models (Fig. 3n). Generally, the highest Max von Mises stresses in the three types of implants were recorded in lateral force application (Fig. 3n). In all loading scenarios for the three models, the Max von Mises stresses were located at the implant body neck area corresponding to the cortical bone level (Fig. 5b,d,f).

**Fig. 6:**
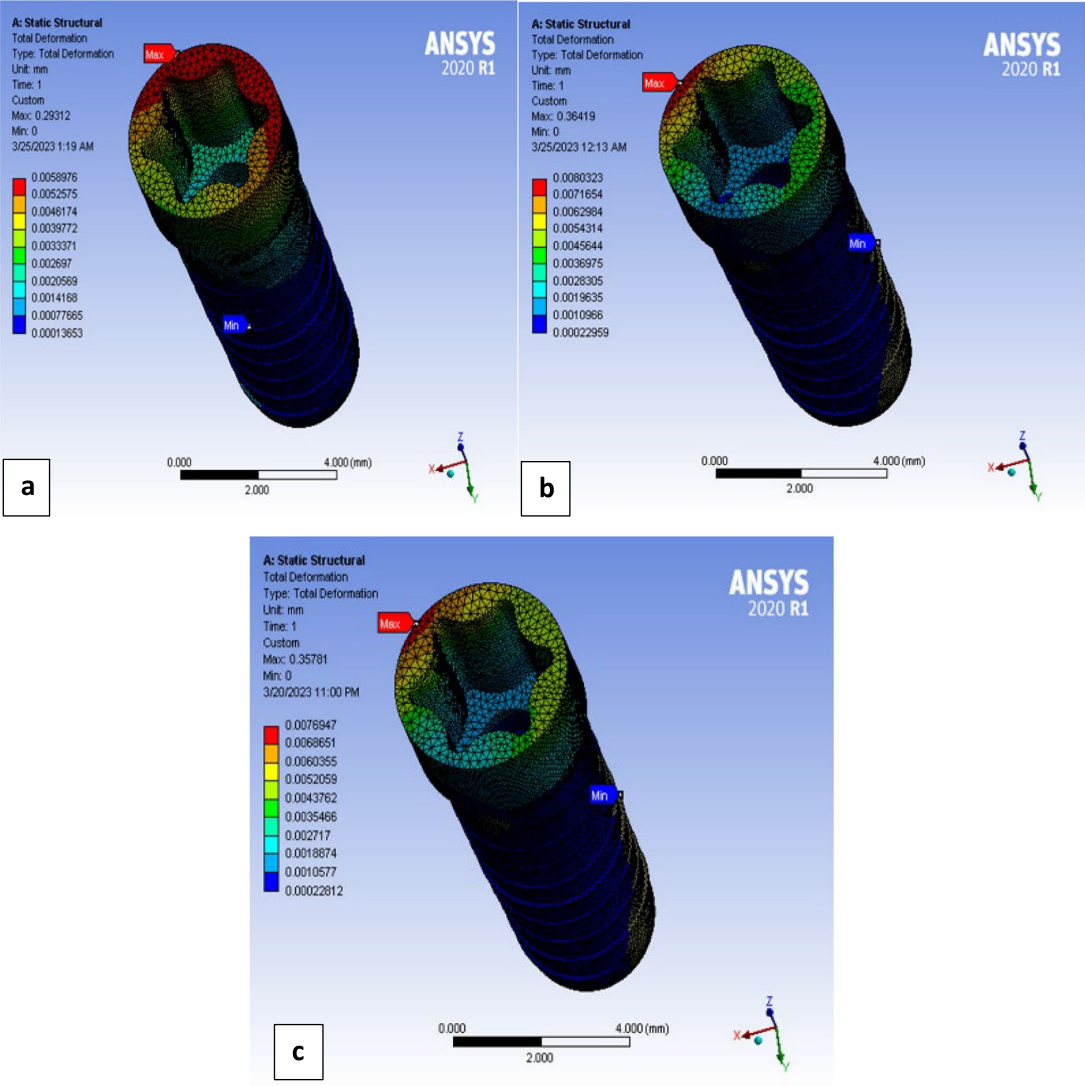
Total deformation of implant body under oblique loading in case of: **a** Ti, **b** PEEK, **C** PEKK

Distribution of deformation patterns along the length of implant body was relatively more uniform in titanium implant than that of polymeric ones. As polymeric implants showed concentrated deformation pattern at coronal parts of the implant body (Fig. 6).

### Cancellous (spongy) bone

It received total deformation of less than 4 microns in all models. It was observed that total deformation values at spongy bone in case of using polymeric implants (PEEK or PEKK) were nearly identical to those of titanium alloy model, in all loading scenarios. Regarding Max von Mises stresses, polymeric models showed lower values in peri-implant spongy bone, than corresponding values in titanium model, especially after lateral and oblique forces applications. Max von Mises stresses values of spongy bone were lower in polymeric models (PEEK or PEKK) than titanium model, by about 0.4 – 0.6 MPa in case of lateral and oblique forces application (Fig. 3p,q).

### Cortical bone

It received total deformation of less than 5.5 microns in all models, with minor sensitivity to implant material in case of lateral and vertical loading scenarios. Ti model showed slightly higher deformation values than polymeric models in case of oblique loading (Fig. 3o).

## Maximum equivalent strain of bone

**Fig. 7:**
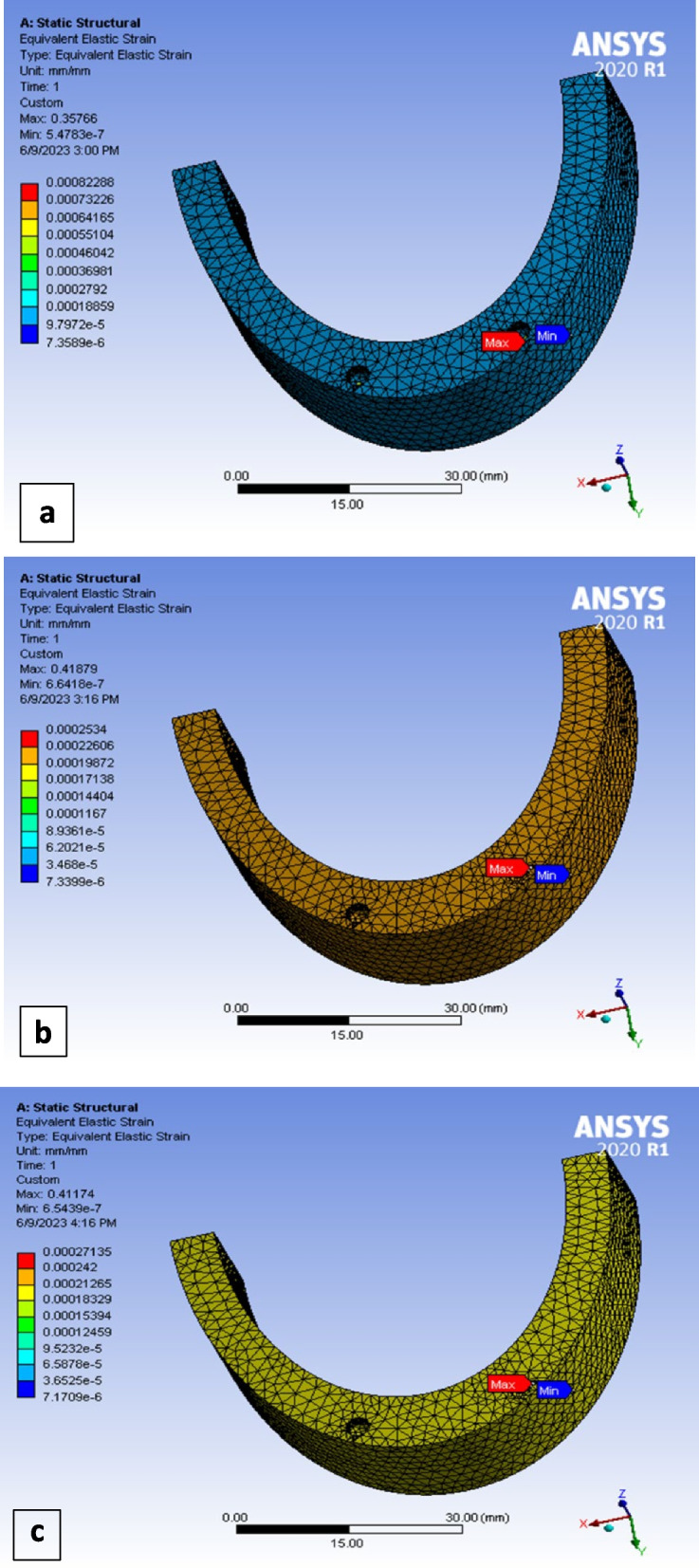
Maximum equivalent strain at spongy bone under lateral loading of: **a** titanium model, **b** PEEK model, **c** PEKK model

Max equivalent strain values of cortical and spongy bone were extracted for the three models (Titanium alloy, PEEK and PEKK) (Fig. 7). They were demonstrated in Table 3 for cortical bone values, and Table 4 for spongy bone values.

**Table 3:** Max equivalent strain value in peri-implant cortical bone for the three case models represented by (ε)

Cortical bone	Titanium model	PEEK model	PEKK model
Vertical loading	0.0015419	0.0017695	0.0017991
Lateral loading	0.0014995	0.0024585	0.002492
Oblique loading	0.0010773	0.0023947	0.00248

**Table 4:** Max equivalent strain value in peri-implant spongy bone for the three case models represented by (ε)

Spongy bone	Titanium model	PEEK model	PEKK model
Vertical loading	0.00053444	0.00048057	0.0004856
Lateral loading	0.00082288	0.0002534	0.0002713
Oblique loading	0.000677	0.00036835	0.0003836

## Maximum and minimum principal stresses of cortical bone

**Fig. 8:**
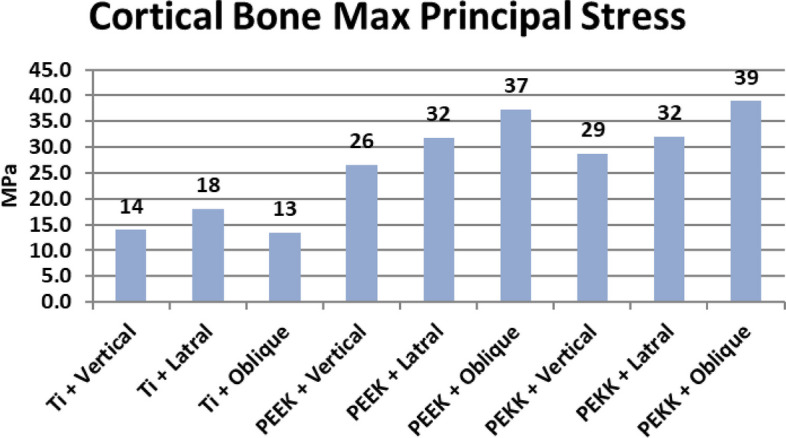
Graphical representation of Maximum principal stresses in cortical bone around implants in the three models (Titanium, PEEK and PEKK)

**Fig. 9:**
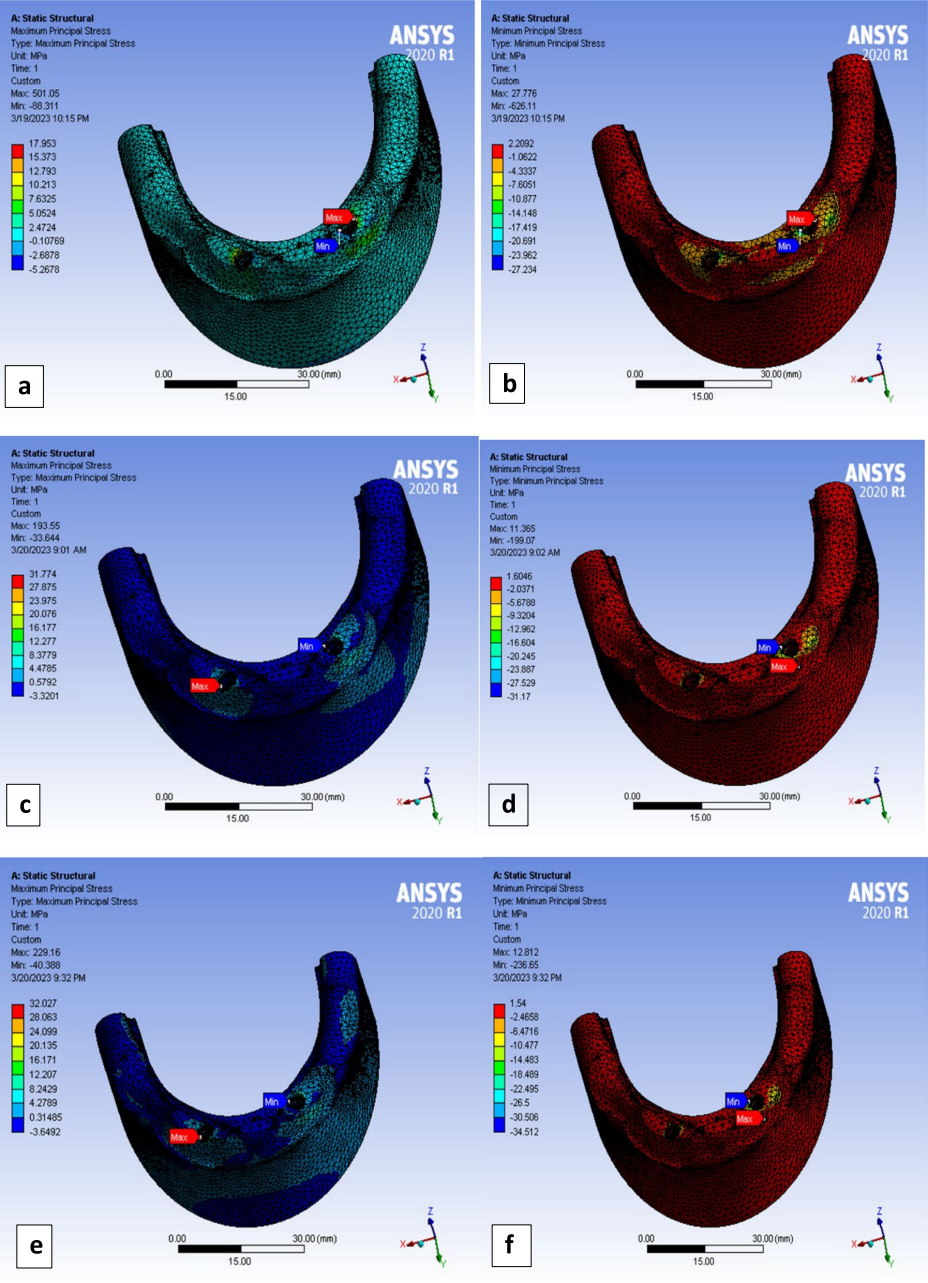
Maximum principal stresses at cortical bone under lateral loading of: **a** titanium model, **c** PEEK model, **e** PEKK model. Minimum principal stresses at cortical bone under lateral loading of (**b**) titanium model, **d** PEEK model, **f** PEKK model

Due to the brittle nature of cortical bone [37], its maximum and minimum principal stresses values were extracted in the three models, for the sake of failure theory analysis (Figs. 8 and 9).

Extracted values of maximum and minimum principal stresses (MaxPS and MinPS) for peri-implant cortical bone were listed in Table 5, it was observed that values of MaxPS around PEEK and PEKK implants were nearly double those of titanium implant, during vertical and lateral loading scenarios. While, in oblique loading they were more than double those of titanium implant.

**Table 5:** Maximum and Minimum principal stress values for cortical bone in the three models of Ti, PEEK and PEKK represented by (MPa) unit

	**Unit**	**MPa**	**MPa**
	***Cortical bone***	**Maximum Principal stress**	**Minimum Principal stress**
R*1	***Ti model Vertical loading***	13.9480	27.5550
***R2***	***Ti model Lateral loading***	17.9530	27.2340
***R3***	***Ti model Oblique loading***	13.3470	17.9580
***R4***	***PEEK model Vertical loading***	26.4770	27.4170
***R5***	***PEEK model Lateral loading***	31.7780	31.1700
***R6***	***PEEK model Oblique loading***	37.3450	29.0910
***R7***	***PEKK model Vertical loading***	28.7630	28.3630
***R8***	***PEKK model Lateral loading***	32.0270	34.5120
***R9***	***PEKK model Oblique loading***	38.9360	30.1570

^*^R  run

By analyzing data of minimum principal stress (MinPS) values in peri-implant cortical bone, it was observed that those of polymer models were nearly in the same range of titanium model values during vertical loading. However, in case of lateral and oblique loadings, (MinPS) values in peri-implant bone of polymer implants showed higher values, especially in oblique loading which were in range of (29–30 MPa) around polymer implants compared to about 17 Mpa in case of titanium implants (Table 5).

## Discussion

The current study investigated high performance thermoplastic polymers from PAEK family as a substitute for titanium alloy as a monolithic implant prosthesis, trying to avoid problems related to the use of titanium alloy, as metal sensitivity, toxicity, and metallic taste as well as stress shielding to bone which can cause bone resorption with time [38, 39]. Pure PEEK and PEKK polymers were chosen as they show better stability and preservation of nanomechanical properties than reinforced thermoplastic polymer composites. Moreover, their composites were subjected to degradation of mechanical properties after aging in saliva with pH fluctuations due to increased spacing between polymer chains, making their long term stability questionable [40].In this study, the possibility of avoiding the stress shielding effect was evaluated, following a monolithic principle in each model, aiming to get more uniform stress distribution in peri-implant bone than that of titanium alloy material.

Regarding overdenture supra-structure, the difference between the three models was in the amount of total deformation in the acrylic material, which was higher in polymer models (PEEK and PEKK). The highest values were observed in lateral and oblique forces. This could be related to the low elastic modulus of polymers (PEEK and PEKK) [31], which led to more deformation of PEEK and PEKK ball attachments than that of Titanium attachment, this higher deformation of polymer ball attachments led to a higher deformation of acrylic overdenture retained by them, especially after application of forces with horizontal components (lateral and oblique).

The greater deformation of acrylic overdenture in polymer models (PEEK and PEKK) caused greater deformation in mucosa than that in case of Ti model. Also, greater Maximum (Max) von Mises stresses in mucosa (0.55—0.57 MPa) were observed in polymer models, especially after application of vertical and oblique forces, approaching the pain threshold of mucosa which is in the range of (0.63–1.2 MPa) [41]. Accordingly, it can be concluded that the clinical use of polymer mandibular overdenture models (PEEK or PEKK), may cause mucosal pain and inflammation, especially if mastication forces exceeded 60 N, as it is expected for complete edentulous patients to have higher mastication force values after using implant supported overdenture [42].

These results were in accordance with Yomna et al. [20], which demonstrated that using PEEK material instead of titanium in the fabrication of “All on four” hybrid prosthesis, increased the stress values of mucosa. Meanwhile, Chen et al. [43] demonstrated that using PEEK as a removable partial denture framework induced higher mucosal stresses than in case of Co-Cr or Ti alloy frameworks.

Regarding stress and deformation analysis of prosthetic units (matrix, ball attachment, gingival former and implant body) in the three models, it was evident that polymer materials (PEEK and PEKK) prosthetic components showed lower Max von Mises stresses and higher total deformation values than corresponding Ti units. These results were explained by the lower modulus of elasticity of PEEK and PEKK than that of Titanium alloy [17]. The results were in accordance with other research, which used PEEK material instead of titanium, for making “All on four “ parts of mandibular hybrid prosthesis, accordingly maximum von Mises stresses on bar, copings, screws, abutments, and implants were reduced [20]. Haron F et al. also evaluated the stress distribution by FEA, in the “All-on-4” prosthesis by using different materials combinations and different opposing arch materials, they found that PEEK framework had lower stress values when compared to titanium one [16].

All PEKK prosthetic components (matrix, ball attachment, gingival former and implant body) showed higher Max von Mises stresses and lower deformation values than PEEK components. This can be explained by the higher rigidity of PEKK than PEEK [17], which may be related to the extra ketone group in PEKK chemical structure [44].

The highest Max von Mises stresses in titanium prosthetic elements were in gingival former under oblique load (333 MPa), which was in accordance with results of other studies demonstrating the effect of oblique force. In the three models of this study, Max von Mises stresses in the implant body were always located at the neck of implant, under different loading conditions [4, 19, 45].

Generally, Dental biomaterials are under dynamic cyclic loading in oral cavity. Therefore, biomechanical evaluation of implant biomaterials does not depend only on comparing Max von Mises stresses with yield strength or ultimate strength values, which were determined by static mechanical tests, but also fatigue failure must be taken into consideration [46]. Fatigue failure may occur at low stress values, lower than ultimate tensile strength or even lower than yield strength [33, 47].

By analysis of Max von Mises stresses in titanium prosthetic elements, it was evident that they did not exceed the yield strength or the fatigue limit of Titanium alloy Grade V, which are about 860–870 MPa and 500 MPa respectively [10, 19]. On the other hand, by extracting stress analysis data of polymer materials (PEEK and PEKK), the highest Max von Mises values were located at PEEK and PEKK gingival formers, which showed 71 and 80 MPa respectively, with lateral loadings.

Endurance limit depends on many factors such as: PEEK microstructure, crystalline structure percentage, fabrication method and the rate of cooling, thus estimation of endurance limit is complicated, with other factors affecting the service life of PEEK as aging process, type of force and the range of service temperature, which lead to what is called yield stress evolution [48].

In a study by Pastukhov LV et al., it was found that endurance limit of compression molded PEEK at temperature of 23 ^o^ C was approximately 70–80 MPa [48]. The fatigue limit of 3D printing PEEK filament materials was estimated to be approximately 59 MPa [49]. Estimation of factor of safety (FOS) for PEEK prosthetic elements depended on ratio between PEEK yield strength (95 MPa) [15] and highest Max von Mises stresses in PEEK prosthetic elements (gingival former, implant body), which was found to be in the range of 1.3 to 1.6 [50].

In a study of long term failure of carbon/PEKK thermoplastic composites by cyclic loading, failure occurred at 80 MPa after 10^4^ seconds in case of 10 Hz frequency [50]. Taking into consideration the tensile strength of PEKK (about 115 MPa) [17] and Max von Mises stresses of PEKK components in this study (80 MPa in gingival former, 64 MPa in implant body), it was evident that low or unsafe FOS values existed for PEKK prosthetic elements [19].

Mechanical properties of thermoplastic polymers such as PEEK are affected by various factors such as temperature and strain and stress rate, due to the viscoelastic behavior of PEEK [51].

Polymer implants are exposed to heat fluctuations during implant placement in mandibular bone, especially in high density bone, and during daily beverage and food consumption. Accordingly, High factor of safety is crucial for the long-term success of semi-crystalline thermoplastic polymers as PEEK or PEKK when they are used in load bearing areas [52].

By reviewing the fatigue limit data of PEEK and PEKK in literature, and low FOS of the two polymer materials in this study, it was evident that the two polymer overdenture models in this study, would carry the risk of yielding or fatigue failure of prosthetic components (gingival former and implant body) under lateral and oblique loading conditions. Accordingly, the first null hypothesis was rejected by the authors [53].

Frost HM [54] proposed a model, which depends on the strain magnitude ε to explain the failure theory of bone, “Frost's mechanostat theory” [55] stated that strain can be directly correlated with the amount of induced stress. In oral cavity, the amount of strain induced in bone depends on many factors such as: occlusal loads, line of force action, and mechanical properties of the bone. According to Frost's theory there are four micro-strain zones, with correlated mechanical adaptation: (a) disuse atrophy, (b) steady state, (c) physiological overload, and (d) pathological loading [37].

In the current study, the highest maximum equivalent bone strain values were always located in the cortical bone, at implant neck region. this observation was the same in the three models, and are consistent with results of other studies [4]. Maximum equivalent cortical bone strain values observed around polymer (PEEK and PEKK) implants were higher than those around titanium implant, especially in oblique and lateral loading scenarios. However, the opposite was observed in the case of spongy bone, as lower maximum equivalent strain values were observed in spongy bone around polymer implants than those around Ti implants. According to our results, peri-implant cortical bone in polymer models showed the highest maximum equivalent strain values of 0.0025, but they did not reach the pathological limit, as the known pathological bone damage occurs when ε > 0.0035. [4, 20].

Due to the brittle nature of cortical bone, several authors proposed that the maximum normal stress (MNS) theory would be more accurate for the prediction of cortical bone failure [56]. According to MNS theory, if one of the three principal stresses became equal or exceeded the strength, failure would occur. MNS theory predicts that failure occurs whenever σ_1_ ≥ S_ut_ (ultimate tensile strength) or σ_3_ ≤  − S_uc_ (ultimate compressive strength) [56]. By extracting data of maximum principal stresses (MaxPS) in peri-implant cortical bone, values of MaxPS around PEEK and PEKK implants were nearly double those of titanium alloy implant, during all loading scenarios. The highest value of MaxPS in cortical bone (39 MPa) in case of oblique loading of PEKK implant did not exceed 40% of cortical bone tensile yield strength (about 100 MPa) [20].

Cortical bone around polymer implants presented higher minimum principal stresses (MinPS) values than those of Ti model in lateral and oblique loadings. However, highest values of MinPS in model of polymer implants (34 MPa) does not exceed 25% of cortical bone compressive yield strength (about 140 MPa) [20].

Regarding Max von Mises stresses of peri-implant spongy bone, polymer implants showed lower values than those of titanium alloy. PEEK implant showed minimum Max von Mises stresses in spongy bone during lateral loading. Max von Mises stresses of spongy bone in both titanium and polymer models did not exceed its tensile or compressive yield strength. The same trend was recorded by analysis of maximum equivalent strain in spongy bone, which did not exceed the physiological limit [20]. Knowing that spongy bone tension and compression yield strengths are in the range of 1.75 MPa and 2.25 MPa respectively [57–59].

Another interesting finding in this study was that polymer implants showed lower maximum equivalent micro-strain values in peri-implant spongy bone than those of titanium. As in the case of titanium model it was 0.0008 during lateral loading, and the corresponding maximum value in polymer models was 0.000485. This finding could be correlated to the deformation patterns of polymer implants observed in this study. By analysis of implant deformation images, it was evident that polymer (PEEK or PEKK) implants showed concentrated and high deformation patterns on the outer surface of their collar parts which were higher than those of Ti implant, while the apical parts of polymer implants showed lower deformation values than their titanium counterparts. As a result, titanium implants showed more uniform deformation pattern along its length than polymer ones, which could explain the lower stresses and strain values transferred to spongy bone around polymer implants. The non-uniform deformation pattern of polymer implants could be attributed to their lower modulus of elasticity [17], which made the coronal parts of polymer implants located above crestal bone level show more deformation than the lower two third parts.

Spongy bone is well adapted to accept stress [60], and studying of the equivalent strain values generated in it is considered by some authors as an evaluation method of stress-shielding effect [61], which follows Frost’s mechanostat theory [37]. By reviewing values of equivalent strain generated in peri-implant spongy bone in titanium model, they were < 0.001 ε in all loading scenarios**,** which was in the disuse atrophy range [61], moreover The two polymer models showed corresponding lower equivalent strain values. Accordingly, polymer implants could cause the same stress shielding effect as titanium.

Regarding the high stress values concentration at implant neck level in the three models, this could be due to the higher modulus of elasticity of cortical bone making it more resistant to deformation than spongy bone [45]. Therefore, cortical bone acts as a fulcrum under oblique force loading, and absorbs higher stresses and loads than spongy bone [37]. In the current study, the higher deformation of polymer (PEEK and PEKK) implants resulted in more bending and more stress concentration in cortical peri-implant bone, especially in case of oblique loading, which generated high maximum principal stress values.

The results of current study were consistent with the results of Schwitalla et al., as they found that pure PEEK implant caused highest Max von Mises stresses in peri-implant cortical bone when compared to Ti [15]. It is expected that polymer implants would show more stress concentration in per-implant cortical bone if mastication force exceeded 60 N, which is expected from overdenture wearer, who could develop mastication forces > 100 N with time, after using implant supported mandibular overdenture [42].

As polymer implants caused higher von Mises stresses in cortical bone, and lower strain values in spongy bone. Therefore, using polymer (PEEK or PEKK) prosthetic components did not overcome the problem of stress shielding effect caused by titanium implants [62]. Accordingly, the second null hypothesis was rejected by the authors.

Strain and stress distribution in peri-implant spongy bone is multifactorial. The health state of overlying cortical peri-implant bone is crucial for strain and stress distribution at underlying spongy bone. Marginal cortical bone resorption could transfer higher strain values to spongy bone, making the value of further future research evaluating stress shielding of PEEK and PEKK implants supporting mandibular overdenture in bone with marginal bone loss [61].

Incomplete Bone implant contact (BIC) is one of disadvantages related to both PEEK and PEKK implants [63, 64]. Bone density and its quality has an important effect in von Mises stresses distribution of peri-implant bone. As bone density decreases from D1 to D4, the generated von Mises stresses in peri-implant bone increase and progress more apically. Decreased BIC (osseointegration) can lead to an increase in the progression of stresses to the apical direction [65]. More research studies are needed to evaluate the overdenture models of the current study with combinations of different bone densities (D1 to D4) and different degrees of BIC.

Results of this study could have important clinical relevance, represented by the expected mucosal pain and inflammation when polymer overdenture models are used. Possible Fatigue failure of polymer implant body or attachments which could lead to malocclusion or retrieval surgery of broken implants or attachment [66]. Also findings of current study necessitates decreasing the magnitude of non-axial forces, through clinical and laboratory steps, such as occlusal adjustment and selecting appropriate occlusal scheme, to decrease lateral component of occlusal forces, especially when utilizing polymer prosthetic components [67, 68].

FEA is a widely used biomechanical evaluation tool in dentistry, but it has some limitations, as it depends mainly on the type and value of numerical data supplied to the software. In this study, it was assumed that the bone is a linear elastic, isotropic, and homogeneous material subjected to axial, lateral and oblique stresses only. Additionally, it was assumed that 100% osseointegration existed between implant and bone. However, these assumptions did not represent in vivo real clinical situations of synchronized muscular movements or the histological properties of bone [69, 70–72].

## Recommendations

1. Biomechanical research studies are needed to evaluate using PEEK and PEKK implants for the support of mandibular overdentures, but with different implant dimensions (length, diameter), different number and distribution of implants, other than those used in this study.
2. More studies are needed to evaluate the overdenture models of the current study with combinations of different bone densities (D1 to D4) and different degrees of bone implant contact.
3. Future research should modify the testing conditions of PEKK and PEEK as biomaterials for implant-supported overdenture to avoid oversimplified simulation of forces applied only in vertical, horizontal and oblique directions.
4. Integrating the impact of muscle movements and their synchronization during chewing into modeling is necessary for achieving more accurate results.
5. Stress analysis should be conducted under dynamic load to determine if higher stresses might be generated at implant-bone interface.
6. The effect of different numbers and distributions of polymer implants supporting overdenture on stress shielding should be investigated.

## Conclusions

Within limitations of this study, it can be concluded that:

- Using two PEEK or PEKK implants in the canine region to support mandibular overdenture using gingival formers, ball attachments and matrices from the same implant polymer material, could cause the following:

1- Generation of high Max von Mises stresses under non-axial forces in polymer gingival former and implant body with low FOS values, consequently fatigue failure of polymer prosthetic components is expected.

2- Low strain and stress values could be induced in peri-implant spongy bone that are lower than those generated by using titanium alloy. Consequently, stress shielding effect and disuse atrophy of peri-implant bone are expected by using polymer prosthetic components.

## Data Availability

The raw data required to reproduce these findings is available upon reasonable request from the corresponding author. The processed data required to reproduce these findings is available upon reasonable request from the corresponding author.
