# Correction: Influence of different polymeric materials of implant and attachment on stress distribution in implant-supported overdentures: a three-dimensional finite element study

**DOI:** 10.1186/s12903-025-06933-z

**Published:** 2025-09-17

**Authors:** Sherif Elsayed, Yousra Ahmed, Mohamed I. El-Anwar, Enas Elddamony, Reem Ashraf

**Affiliations:** 1 Al-Ryada University for Science and Technology, Sadat City, Menoufia Egypt; 2 Department of Prosthetic Dentistry, Removable Prosthodontics Division, Faculty of Dentistry, King Salman International University, El Tur, South Sinai Egypt; 3 Mechanical Engineering Department, National Research Centre (NRC), Dokki, Giza, Egypt; 4 Department of Prosthetic Dentistry, Biomaterials Division, Faculty of Dentistry, King Salman International University, El Tur, South Sinai Egypt; 5 Faculty of Dental Medicine, Suez Canal University, Ismailia, Egypt

**Correction: BMC Oral Health 25, 166 (2025)**

**https://doi.org/10.1186/s12903-025-05440-5**

In this article [1], the author would like to add another affiliation “Faculty of Dental Medicine, Suez Canal University, Ismailia, Egypt” for the co-author Enas Elddamony.
